# Exploring the Therapeutic Potential of Ganoderic Acid A Against Inflammatory Bowel Disease Based on Network Pharmacology, Molecular Docking, and Intestinal Organoid Validation

**DOI:** 10.3390/ijms27135698

**Published:** 2026-06-24

**Authors:** Min Cai, Manhui Sun, Kecheng Li, Zhenzhen Wang, Jianwei Mao, Ruyi Sha

**Affiliations:** 1 School of Biological and Chemical Engineering, Zhejiang University of Science and Technology, Hangzhou 310023, China; 2 Zhejiang Provincial Key Laboratory for Chemical & Biological Processing Technology of Farm Product, Hangzhou 310023, China

**Keywords:** inflammatory bowel disease, ganoderic acid A, ferroptosis, bioinformatics

## Abstract

Inflammatory bowel disease (IBD) poses a significant global health burden with rising incidence, particularly in Asia. This study employed an integrative network pharmacology approach combined with molecular docking to elucidate the therapeutic mechanism of ganoderic acid A (GAA) against IBD. Potential GAA targets were retrieved from pharmacogenomic databases, while IBD-related genes were curated from OMIM and GeneCards databases. Weighted gene co-expression network analysis of IBD transcriptomic datasets (GSE38713, GSE126124) identified disease-associated modules, with the yellow module exhibiting the strongest positive correlation. Functional enrichment analyses demonstrated significant involvement of overlapping targets in lipid metabolism, the inflammatory response, and the mitogen-activated protein kinase (MAPK) signaling cascade pathway. We identified 14 IBD-GAA-ferroptosis-related genes and 54 key module genes. Intersection analysis revealed 5 overlapping targets, including tumor necrosis factor-α(TNF-α), peroxisome proliferators-activated receptor γ (PPARγ), MAPK14, phosphatidylinositol-4,5-bisphosphate 3-kinase catalytic α (PIK3CA), and Caspase 3 (CASP3). Molecular docking confirmed high-affinity binding of GAA to these targets, with binding energies ranging from −7.3 to −10 kcal/mol. Crucially, experimental evaluation demonstrated the pivotal role of GAA in alleviating disease pathology. GAA treatment suppressed the significantly elevated levels of TNF-α and p-MAPK14 in the organoids using a cytokine/LPS-induced IBD model. These findings collectively suggest a potential involvement of GAA in pathways associated with ferroptosis regulation, although direct experimental evidence for ferroptosis markers remains to be established. The observed multi-target effects on immune regulation and cellular proliferation/differentiation provide a foundation for further mechanistic investigation.

## 1. Introduction

Inflammatory bowel disease (IBD) represents a chronic relapsing inflammatory condition that primarily involves the ileum, colon, and rectum. It had a predilection for affecting young to middle-aged adults [1]. The global incidence of IBD has been escalating, with prevalence rates exceeding 0.6% in Western and developing countries [2]. Its pathogenesis is multifactorial, involving genetic predisposition, environmental triggers, intestinal microbiota dysbiosis, and dysregulation of the immune system [3]. Owing to the chronic nature of this disorder, patients require long-term and often lifelong management, which imposes a substantial psychological burden and significantly compromises their quality of life.

Emerging evidence highlights a critical role for ferroptosis—a non-apoptotic, iron-dependent form of regulated cell death driven by lipid peroxidation in IBD progression [4,5]. Intestinal epithelial cells (IECs), vital for maintaining mucosal barrier integrity, are particularly susceptible to ferroptotic injury under inflammatory conditions. Dysregulated iron metabolism was evident in IBD patients as elevated serum ferritin and reduced transferrin saturation [6]. It promotes labile iron accumulation in the gut mucosa, catalyzing reactive oxygen species (ROS)-mediated lipid peroxidation [7]. Preclinical studies show that inflammatory cytokines (e.g., TNF-α, IFN-γ) downregulate the cystine/glutamate antiporter SLC7A11, depleting intracellular glutathione and exacerbating lipid peroxidation in IECs as a hallmark of ferroptosis [8]. This process disrupts epithelial barrier function, allowing bacterial translocation and amplifying mucosal inflammation to create a vicious cycle in IBD pathogenesis. Several studies highlight emerging therapeutic strategies targeting ferroptosis in IBD, including targeting mitochondrial pathways to mitigate disease severity [9]. However, a key gap remains: few natural products with multi-target ferroptosis-modulating effects have been systematically evaluated.

Ganoderic acid A (GAA), a triterpenoid compound derived from the medicinal mushroom *Ganoderma lucidum*, has emerged as a potential therapeutic agent for several diseases by targeting ferroptosis [10,11]. Recent studies demonstrate that GAA exerts protective effects against IBD through dual modulation of iron metabolism and lipid peroxidation [12]. In preclinical models of dextran sulfate sodium (DSS)-induced colitis, GAA treatment significantly ameliorates mucosal damage and reduces inflammatory cytokine production (e.g., IL-6, TNF-α) and restores epithelial barrier function, coinciding with suppressed ferroptosis in intestinal epithelial cells (IECs) [13]. While most evidence currently stems from preclinical models, GAA represents a novel natural compound with translational potential for IBD therapy [14].

In the era of advancing bioinformatics, recent investigations have enabled systematic exploration of the molecular connections between GAA, ferroptosis, and IBD [15]. In this study, weighted gene co-expression network analysis (WGCNA) was employed to identify robust co-expression modules shared between controls and patients with IBD. Subsequent screening of these modules isolated candidate common driver genes, which were functionally annotated using the Kyoto Encyclopedia of Genes and Genomes (KEGG) pathway analyses to characterize enriched biological processes and signaling pathways.

Molecular docking analyses were conducted to characterize the interactions between ganoderic acid A and its predicted target proteins. Originally established as a key computational tool in drug design, this technique was adapted here to investigate the material basis of GAA’s bioactivity by evaluating compound-target binding affinities and interaction modes [16]. The stability of ligand-receptor binding conformations and the likelihood of productive interactions are inversely correlated with binding energy values. Numerous studies have demonstrated the versatile applications of molecular docking in exploring the drug-like properties of natural compounds such as GAA [17]. This strategy not only validates predicted target associations but also provides structural insights into GAA’s mechanism of action, particularly in the context of its reported effects on ferroptosis and inflammatory pathways in IBD [18]. In this study, we employed an integrative strategy combining network pharmacology, WGCNA, and molecular docking to systematically predict the potential targets and mechanisms of GAA against IBD. Unlike conventional computational-only studies, we further performed experimental validation using a human iPSC-derived intestinal organoid model of IBD induced by TNF-α (combined with LPS and IL-1β). This organoid-based approach allowed us to assess the protective effects of GAA on intestinal epithelial damage, proliferation, and key signaling proteins (TNF-α, p-MAPK14, and PPARγ). By integrating bioinformatic predictions with biological validation in a physiologically relevant human intestinal model, our work provides a more comprehensive understanding of GAA’s therapeutic potential and offers a framework for discovering natural product-based interventions for IBD.

## 2. Results

### 2.1. Recognition and KEGG Enrichment Analysis of GAA Targets Against IBD

**Figure 1:**
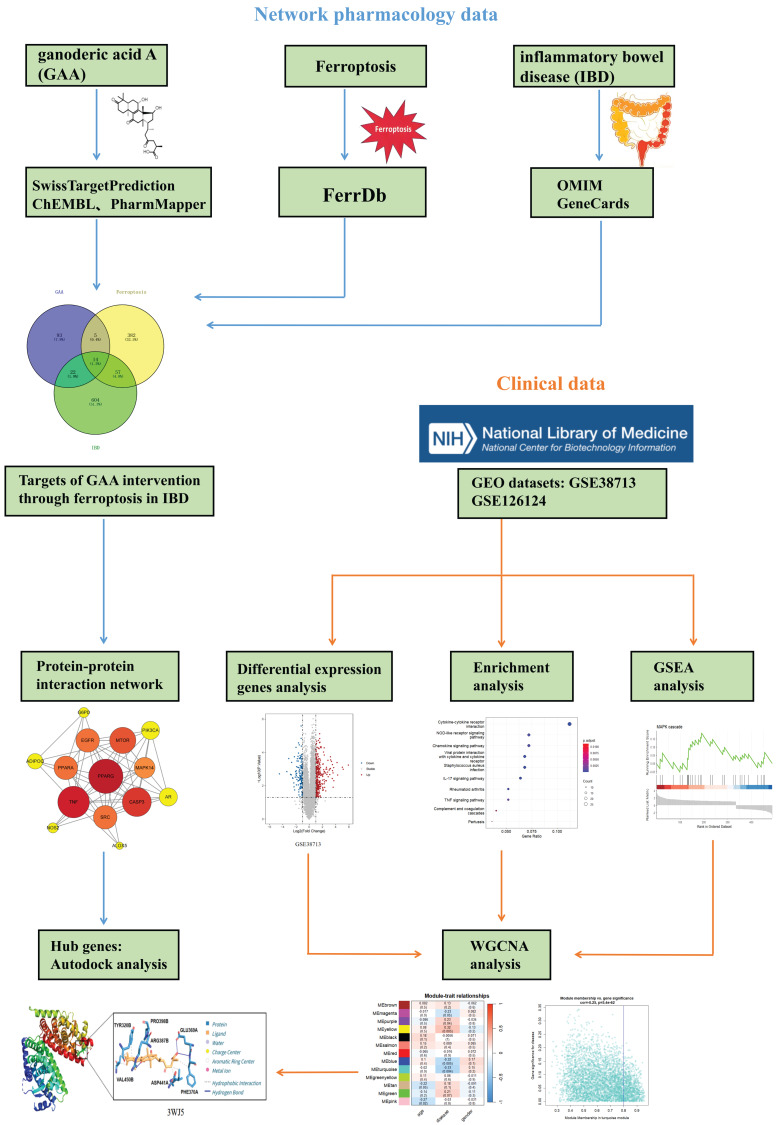
A flowchart illustrating the idea of the project.

**Figure 2:**
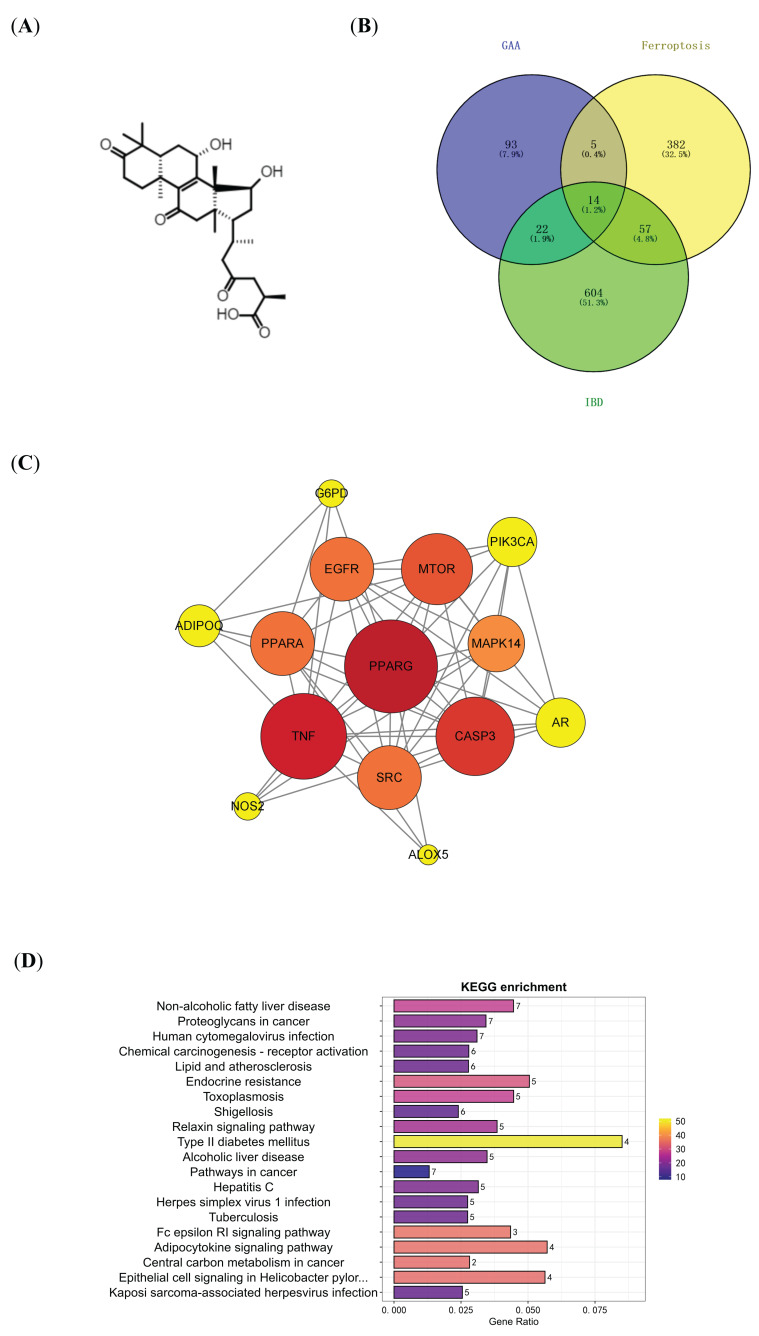
Recognition and KEGG enrichment analysis of GAA targets against IBD: (**A**) The molecular structure of GAA. (**B**) A Venn diagram shows the number of GAA, ferroptosis, and IBD-associated genes. (**C**) Visualization of the PPI network with diosgenin’s targets. The size of the dot represents the degree value of the proteins; lines represent functional associations between the proteins. The yellow nodes represent the degree range of 1 to 10; The orange nodes represent the degree range of 10 to 20; The red nodes represent the degree above 20. (**D**) GAA’s targets involved in the KEGG pathways.

This study explores the potential targets of ganoderic acid A (GAA) for the prevention and treatment of IBD, following the overall approach of network pharmacology analysis (Figure 1). Ganoderic acid A is a highly oxygenated lanostane-type triterpenoid characterized by a tetracyclic scaffold [16]. Its structure incorporates key functional groups, including a carboxylic acid at C-26, a β-hydroxy group at C-3, and carbonyl groups at C-7, C-11, and C-15 positions (Figure 2A). A total of 134 GAA-associated genes, 458 ferroptosis-associated genes, and 697 IBD-related genes were identified via the SwissTargetPrediction, ChEMBL, and PharmMapper databases (Figure 2B, Supplementary Materials S1). Among these, 14 overlapping genes were identified as potential targets of GAA in the context of IBD (Table 1). Subsequently, the overlapping genes were uploaded to the STRING database. The PPI network was constructed using Cytoscape software, version 3.8.2, and the topological properties of all nodes were calculated and visualized (Figure 2C). The target genes were subjected to KEGG enrichment analysis, which was expected to figure out the functional roles in the enriched signaling pathways. The results exhibited the pathways of non-alcoholic fatty liver, cytomegalovirus infection, carcinogenesis, and lipid atherosclerosis (Figure 2D).

### 2.2. Integration and Differential Expression Gene Analysis of the IBD Dataset

Two primary datasets (GSE38713 and GSE126124) comprising samples from IBD patients and healthy controls were retrieved from the GEO database. Following sample integration, batch effect correction, and normalization procedures, a total of 106 intestinal tissue samples (34 healthy controls versus 72 IBD cases) were derived. Principal component analysis after ComBat adjustment confirmed the removal of dataset-specific batch effects while preserving IBD-associated expression variation. Differential expression genes (DEGs) between groups were identified using the limma package with stringent criteria of *p* < 0.05 and |log_2_FC| > 0.5.

**Figure 3:**
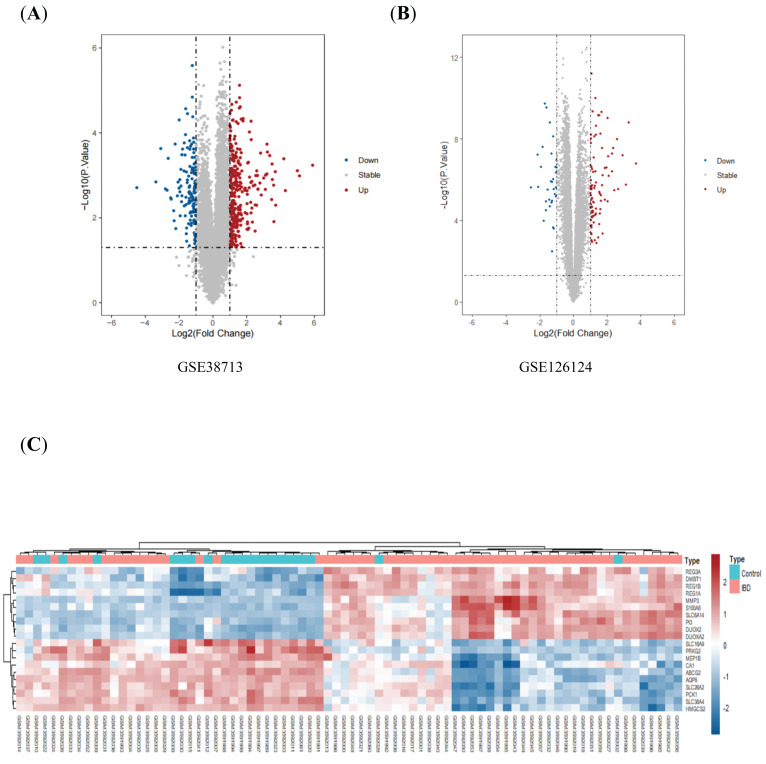
Integration and differential expression analysis of the IBD dataset: (**A**) Volcanic map of DEGs in GSE38713 between IBD patients and controls. (**B**) Volcanic map of DEGs in GSE126124 between IBD patients and controls. (**C**) The top 20 differential DEGs were identified by a heatmap between GSE38713 and GSE126124 between IBD patients and controls.

In intestinal tissue samples, 556 DEGs were detected, including 385 upregulated and 171 downregulated genes (Supplementary Materials S1). Volcano plots (Figure 3A,B) and heatmaps (Figure 3C) were generated to visualize the DEGs identified in these tissue samples, providing a comprehensive overview of gene expression profiles associated with IBD.

### 2.3. Functional Enrichment of IBD-Related Genes in the IBD Dataset

**Figure 4:**
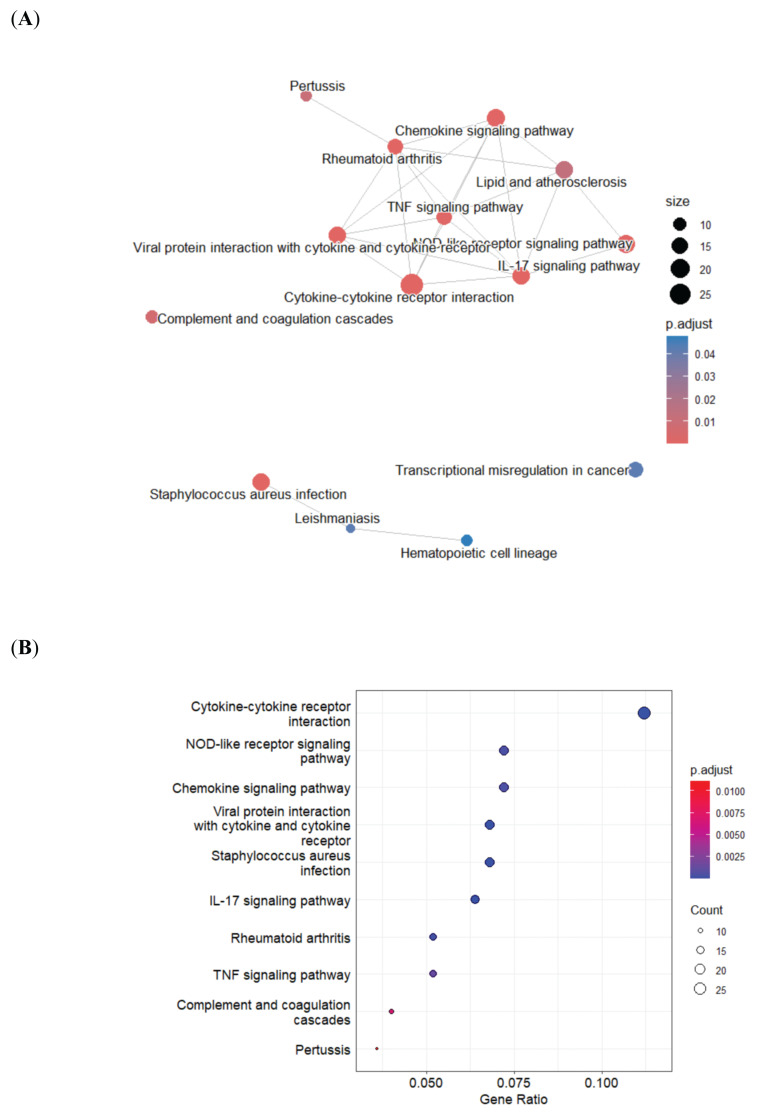
Functional enrichment of IBD-related genes in the IBD dataset: (**A**) PPI network of DEGs. (**B**) Results of the KEGG pathway analysis. The size of the bubble represents the number of involved genes, and the color of the bubble represents the significance of the terms.

To further characterize the functional pathways of IBD-related genes, functional enrichment analysis was performed using the Metascape database. This analysis revealed distinct functional groups among the genes, with the positive regulation of cytokine–cytokine receptor interaction, NOD-like receptor (NLR) signaling pathway, TNF signaling pathways, and lipid atherosclerosis as the most prominent biological processes (Figure 4A,B).

In inflammatory bowel disease (IBD), the NOD-like receptor signaling pathway acts as a critical sensor of intestinal dysbiosis, directly linking microbial cues to pathogenic inflammation. NLRs, particularly NOD1 and NOD2, initiate a cascade that converges on the activation of both the NF-κB and MAPK pathways. This dual activation serves as a master switch: NF-κB drives the transcription of potent pro-inflammatory cytokines, most notably TNF-α, while the MAPK pathway regulates additional cytokine production, cell stress responses, and immune cell activation. The resulting TNF-α then not only exerts its own destructive effects on intestinal epithelial integrity but can further amplify the upstream NLR and MAPK signaling, creating a vicious, self-sustaining inflammatory feedback loop. This synergistic interplay between NLR sensing, TNF-α-mediated tissue damage, and MAPK-driven cellular responses fundamentally disrupts mucosal homeostasis, perpetuating chronic inflammation and tissue injury characteristic of IBD.

### 2.4. GSEA Analysis of the IBD Dataset

**Figure 5:**
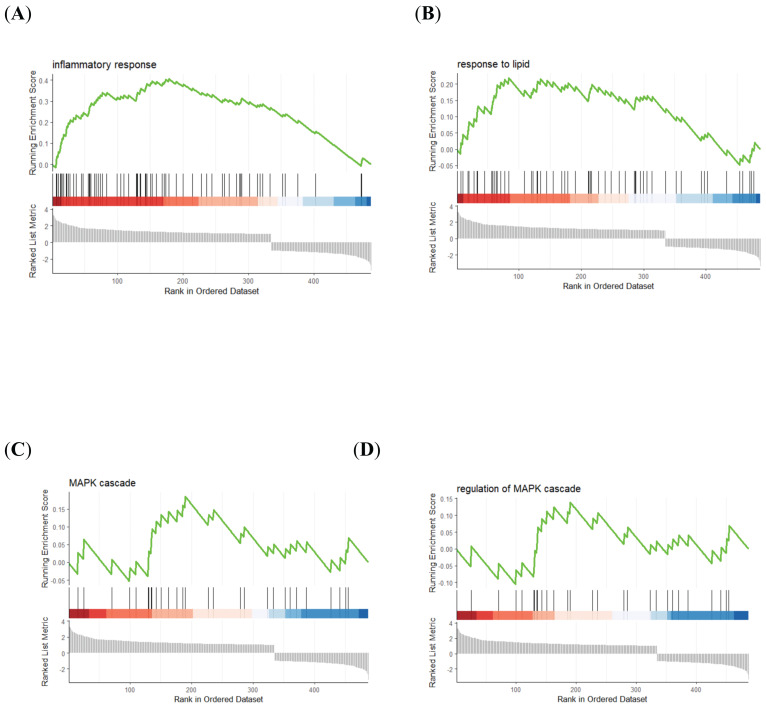
GSEA snapshots of GO term enrichment analysis: (**A**) Inflammatory response. (**B**) Response to lipids. (**C**) MAPK cascade. (**D**) Regulation of the MAPK cascade.

Expression datasets (GSE38713 and GSE126124) were established with GSEA analysis. After that, the key Gene Ontology enrichment and the change in expression level of core related genes were obtained. The results exhibited four important modules, namely inflammatory response, response to lipids, MAPK cascade, and regulation of MAPK cascade (Figure 5A–D). The results indicated that the modules above have significant up-regulation in gene expression levels between IBD patients and controls.

### 2.5. Analysis of WGCNA and Key Module Identification in the IBD Dataset

**Figure 6:**
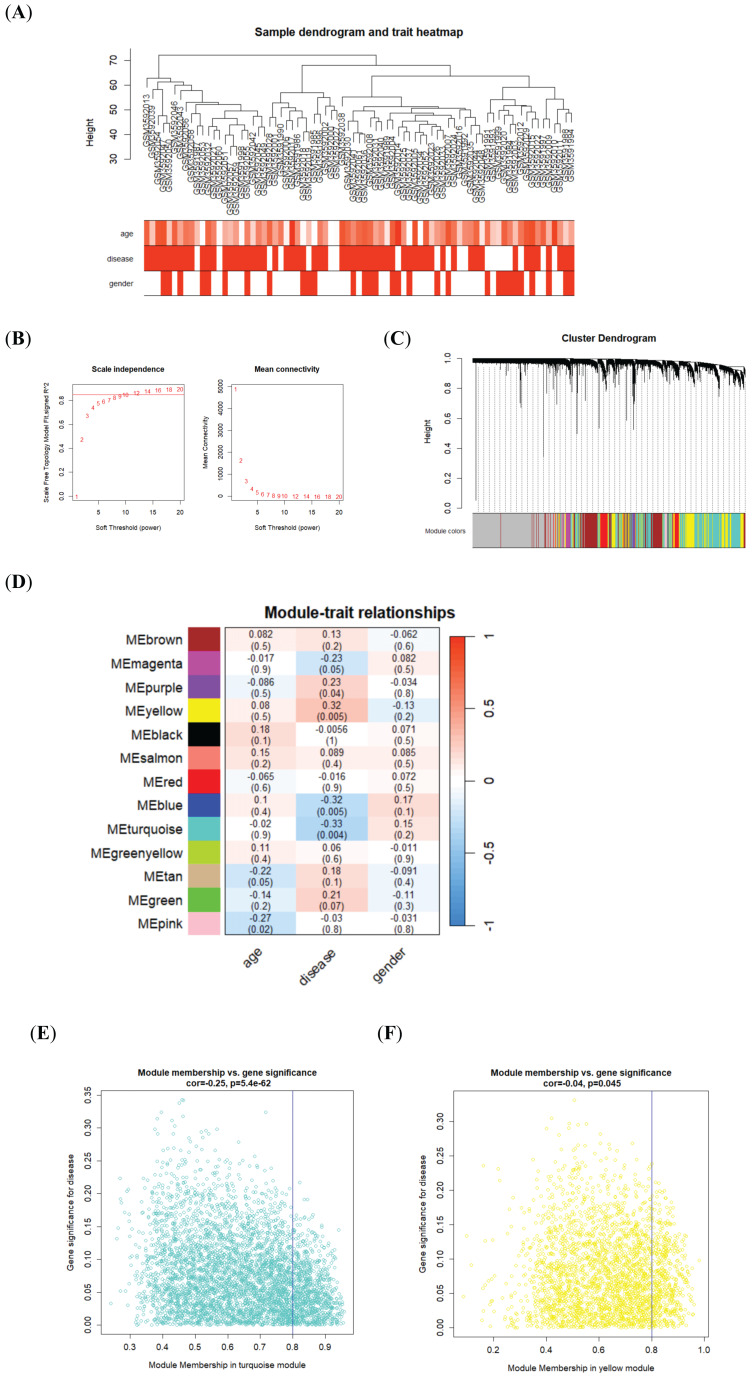
Analysis of WGCNA and key module identification in the IBD dataset: (**A**) Sample dendrogram and trait heatmap of GSE126124. (**B**) Mean connectivity for scale independence and soft threshold. (**C**) Clustering dendrograms of genes in IBD. (**D**) Heatmap revealing the relationship between module-feature genes and IBD status. (**E**,**F**) Correlation plot of turquoise and yellow module members and gene significance in the modules.

**Figure 7:**
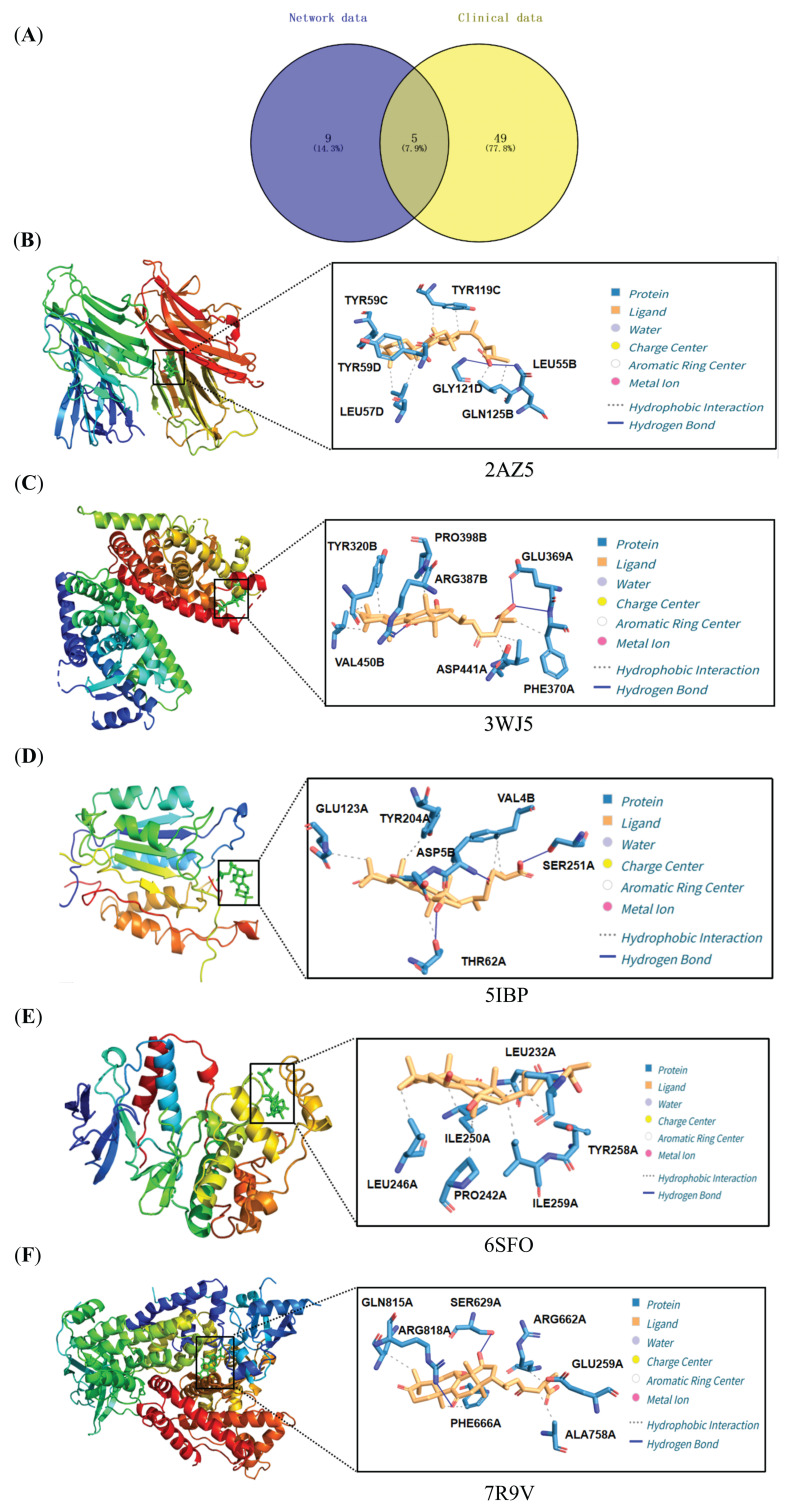
Molecular docking showed the direct binding analysis of GAA to its potential target proteins: (**A**) Venn diagram showing the overlap of 5 common genes between network pharmacology data and clinical data in IBD. (**B**) TNF-α (PDB ID: 2AZ5). (**C**) PPARγ (PDB ID: 3WJ5). (**D**) CASP3 (PDB ID: 5IBP). (**E**) MAPK14 (PDB ID: 6SFO). (**F**) PIK3CA (PDB ID: 7R9V).

To deepen the exploration of key genes in inflammatory bowel disease (IBD), weighted gene co-expression network analysis (WGCNA) was conducted to uncover the most relevant gene modules within IBD samples. After quality control, the sample clustering dendrogram showed that no samples deviate significantly from the main population, indicating that the data quality is high enough to accept the subsequent analysis (Figure 6A). A soft threshold of β = 10 was employed to maintain the average connectivity and topological characteristics of the model, as illustrated in Figure 6B,C. This correlation analysis yielded thirteen distinct color-coded gene modules (Figure 6D) and revealed that the yellow module exhibited the highest association with IBD (r = 0.32, *p* = 0.005). Meanwhile, the turquoise module showed the strongest negative correlation with the disease (r = −0.33, *p* = 0.004). Notably, within the identified modules, a substantial correlation was observed between gene significance (GS) and module membership (MM). Genes with high GS (|GS| > 0.15) and MM (|MM| > 0.8) were prioritized as hub candidates (Figure 6E,F, Supplementary Materials S1). This finding further validated that the module genes are significantly associated with disease pathogenesis. Through WGCNA, we ultimately identified 54 highly related genes that are potential drivers/inhibitors of IBD development (Table 2). Combined with the target genes in Table 1, the 5 overlapping genes (TNF-α, PPARγ, CASP3, MAPK14, and PIK3CA) were selected as hub genes (Figure 7A).

### 2.6. Molecular Docking of Hub Genes

To further elucidate the potential mechanism of GAA, molecular docking simulations were performed to evaluate its binding affinity toward key target proteins. Three-dimensional structures of TNF-α (PDB ID: 2AZ5), PPARγ (PDB ID: 3WJ5), CASP3 (PDB ID: 5IBP), MAPK14 (PDB ID: 6SFO), and PIK3CA (PDB ID: 7R9V) were retrieved from the Protein Data Bank. Binding affinities were calculated using AutoDock Vina between GAA and all five targets (Figure 7B–F). The analysis revealed consistently negative binding energies for each GAA-target complex, ranging from −10 to −7.3 kcal/mol, indicative of predicted favorable binding and suggestive of thermodynamically favorable interactions (Supplementary Materials S3). These thermodynamically favorable interactions strongly implicate the identified targets in the molecular mechanism underpinning GAA-induced IBD regulation. Structural visualizations depicting the lowest-energy binding poses for each complex were generated using Discovery Studio, version 4.0 and PyMol software, version 2.4.0.

### 2.7. GAA Treatment Significantly Alleviates the Cytokine/LPS-Induced IBD Model

**Figure 8:**
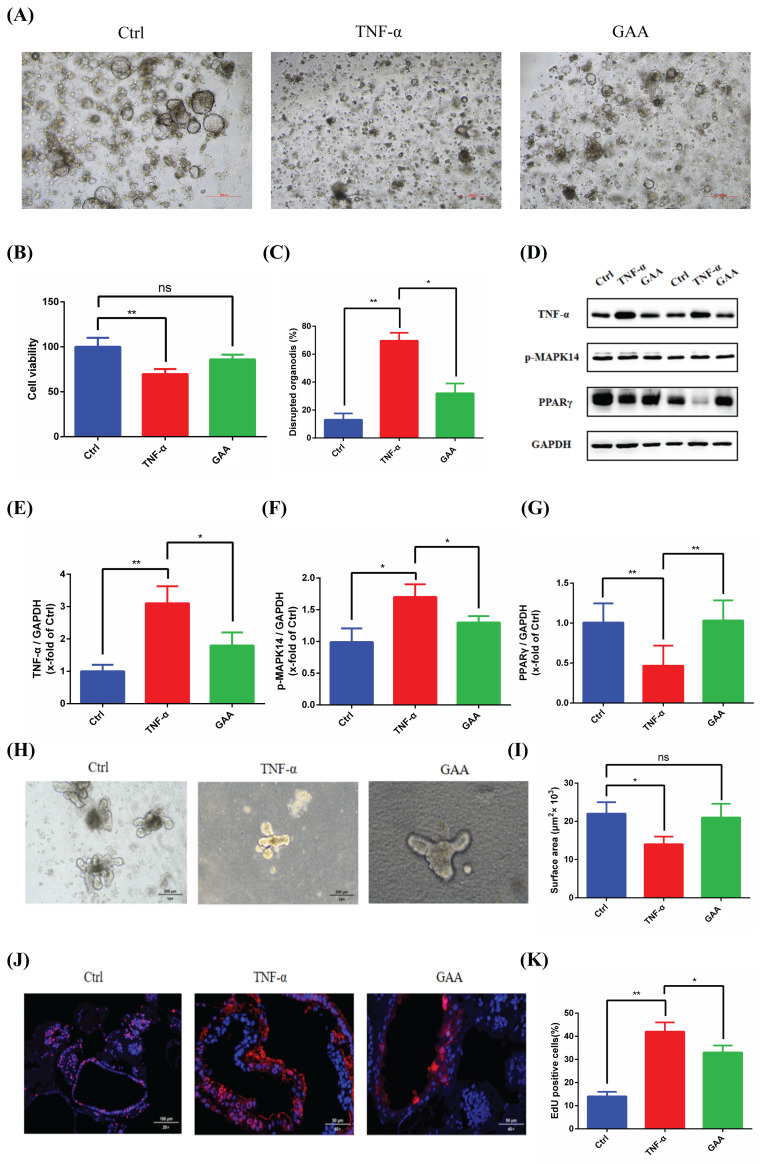
GAA treatment alleviated cytokine/LPS-induced IBD: (**A**–**C**) Morphology (the scale bar represents 500 μm), vitality, and extent of damage of intestinal organoids. (**D**) Immunoblots of proteins isolated from intestinal organoids with control, cytokine/LPS, and GAA treatment. (**E**–**G**) Quantification of relative protein expression from D. (**H**,**I**) Morphology (the scale bar represents 200 μm) and area size of intestinal organoids in different treatment groups. (**J**,**K**) The EdU cell proliferation assay detects proliferation after different treatments (red indicates proliferating cells, the scale bar represents 50 μm) and quantifies the percentage of EdU-positive cells. All error bars are s.e.m. * *p* < 0.05, ** *p* < 0.01 compared with control.

To further verify the findings obtained from the enrichment and molecular docking analysis, iPSC-induced intestinal organoids were established as an inflammatory bowel disease model using 100 ng/mL LPS + 10 ng/mL IL-1β + 10 ng/mL TNF-α treatment (Figure 8A). The results showed that the cell survival rate in the model group significantly decreased, and the organoid damage rate approached 70% (Figure 8B). However, GAA treatment (60 µM) significantly alleviated the damage caused by cytokine/LPS-mediated inflammation (Figure 8C). Western blot analysis revealed that GAA treatment suppressed the significantly elevated levels of TNF-α and p-MAPK14 in the organoids, which were induced by IBD modeling. GAA treatment also increased PPARγ levels, which were supposed to enhance lipid metabolism and anti-inflammatory function (Figure 8D–G). TNF-α and MAPK14 were prioritized because they represent the most upstream and well-documented inflammatory drivers in IBD, with direct clinical relevance (anti-TNF therapy). PPARγ was selected due to its central role in lipid metabolism and anti-inflammatory transrepression, linking our KEGG enrichment findings. CASP3 (apoptosis) and PIK3CA (proliferation/survival) were not experimentally validated due to resource limitations. In addition, GAA treatment prevented the organoids from shrinking caused by cytokine/LPS treatment and maintained the normal morphology of the organoids (Figure 8H,I). EdU cell proliferation assay revealed that the proliferation rate of intestinal epithelial cells was the most prominent in the organoids, indicating severe damage after cytokine/LPS treatment. GAA treatment significantly alleviated this damage (Figure 8J,K).

## 3. Discussion

This study employed a network pharmacology approach, integrating bioinformatics analysis and computational molecular docking to elucidate the potential mechanisms of GAA. GAA is a bioactive dietary compound that has been reported to exert protective effects against IBD. Putative macromolecular targets of GAA were systematically identified using established databases (SwissTargetPrediction, ChEMBL) to characterize its bioactive profile. Comparative analysis revealed that the number of known IBD-associated targets substantially exceeded the predicted targets of GAA. This observation indicates that GAA’s potential therapeutic effects likely arise from its interaction with a specific subset of targets within the broader IBD pathophysiological network.

Inflammatory bowel disease (IBD) represents a chronic condition predominantly affecting young adults and middle-aged individuals. Since the turn of the 21st century, IBD has emerged as a rapidly growing global health concern, with a particularly pronounced increase in prevalence observed across developing nations. Diet constitutes a key modifiable environmental factor implicated in IBD pathogenesis. Epidemiological studies indicate an inverse association between fruit and vegetable consumption and the risk of Crohn’s disease (CD) [19]. Conversely, dietary patterns rich in red meat and fatty fish have been linked to an elevated risk of CD development.

The unique molecular signature of ferroptosis offers promising targets for early IBD diagnosis. Serum and fecal biomarkers of lipid peroxidation, such as malondialdehyde (MDA) and 4-hydroxynonenal (4-HNE), are significantly elevated in IBD patients during subclinical phases, preceding overt clinical symptoms [20]. Lipid peroxidation products such as MDA and 4-HNE disrupt tight junctions through several interconnected mechanisms. For instance, 4-HNE can form Michael adducts with cysteine and lysine residues on occludin and ZO-1, leading to protein redistribution and barrier loss. In addition, 4-HNE suppresses occludin expression at both mRNA and protein levels via TLR4 activation, thereby increasing gut permeability [21]. Elevated MDA levels are also closely linked to ferroptosis: while GPX4 normally inhibits lipid peroxidation to preserve epithelial integrity, dysregulation of this pathway raises MDA and consequently disrupts tight junctions. Beyond these protein-targeted effects, MDA and 4-HNE directly damage membrane lipids by reacting with polyunsaturated fatty acids, which compromises membrane fluidity, promotes ROS production, and indirectly impairs tight junction organization. Collectively, these mechanisms work in concert to mediate tight junction disruption in inflammatory bowel disease.

Iron metabolic parameters, including fecal ferritin and serum hepcidin levels, correlate with disease activity and reflect ferroptotic potential in the intestinal mucosa. Additionally, transcriptomic profiling of intestinal biopsies reveals dysregulation of ferroptosis-related genes (GPX4, ACSF2, and PTGS2) in IBD patients, which can be detected via non-invasive liquid biopsies or stool-derived RNA sequencing [7]. Integrating these biomarkers into a multi-omics diagnostic model may improve early detection, especially in high-risk individuals with a familial IBD history or subclinical gut inflammation. Targeting ferroptosis also holds therapeutic promise, but its role in IBD diagnosis highlights the need for longitudinal studies validating these biomarkers across populations. As our understanding of ferroptosis–IBD crosstalk deepens, these molecular signatures may revolutionize early disease identification, facilitating personalized management strategies to prevent disease progression [22].

Based on in silico predictions, GAA may hypothetically intersect with ferroptosis-related pathways through several candidate molecular nodes. Firstly, GAA might enhance the expression of glutathione peroxidase 4 (GPX4), a pivotal enzyme that neutralizes lipid peroxides and acts as a master regulator of ferroptosis. By potentially upregulating GPX4 at both transcriptional and post-translational levels, GAA could mitigate oxidative stress-induced lipid peroxidation in IECs, particularly under conditions of iron overload [23]. Secondly, the compound may promote the activity of the cystine/glutamate antiporter SLC7A11, which imports cystine for glutathione synthesis, thereby possibly replenishing intracellular antioxidant reserves and reducing sensitivity to ferroptotic stimuli [24]. Additionally, GAA might modulate iron homeostasis by inhibiting transferrin receptor 1 (TFRC)-mediated iron uptake and reducing labile iron pools that drive reactive oxygen species (ROS) production in the gut mucosa [12]. However, these computational inferences remain strictly hypothesis-generating and do not constitute evidence of functional ferroptosis modulation. Integrative analysis of IBD datasets identified 556 consistently differentially expressed genes (DEGs). Subsequent weighted gene co-expression network analysis (WGCNA) pinpointed key modules exhibiting the strongest disease associations. Combining these DEGs with the most relevant WGCNA modules yielded 54 candidate driver genes. Functional enrichment analysis revealed significant overrepresentation of biological processes and pathways, including lipid metabolism, inflammatory response, and MAPK signaling cascades. These findings strongly implicate dysregulation of immune function and lipid metabolic pathways as central contributors to IBD pathogenesis. Furthermore, network pharmacology-based enrichment analysis of the driver genes provided complementary insights. The prioritized core genes identified through this integrated approach were then subjected to molecular docking (AutoDock) for validation.

The intricate pathogenesis of IBD involves a complex interplay of dysregulated immune responses, epithelial barrier dysfunction, and aberrant cellular signaling pathways. Several key molecular players, such as TNF, MAPK14, PIK3CA, CASP3, and PPARγ, occupy central roles. Tumor Necrosis Factor-alpha (TNF) stands as a paramount pro-inflammatory cytokine critically implicated in IBD, driving potent activation of immune cells [4]. It functions by inducing epithelial apoptosis, disrupting tight junction integrity, and promoting the production of other inflammatory mediators like interleukin-1β (IL-1β), IL-6, and chemokines. Its pivotal role is unequivocally validated by the clinical efficacy of anti-TNF monoclonal antibodies (e.g., infliximab, adalimumab) in inducing and maintaining remission in a significant subset of Crohn’s disease and ulcerative colitis patients.

Mitogen-activated protein kinase 14 (MAPK14), also known as p38α MAPK, is directly downstream of TNF receptor signaling. MAPK14 is a serine/threonine kinase that orchestrates cellular responses to stress and inflammation. Activated MAPK14 phosphorylates numerous transcription factors (e.g., ATF2, MEF2C) and kinases, leading to the amplified transcription of pro-inflammatory cytokines, chemokines, and matrix metalloproteinases (MMPs) [25]. Therefore, it may promote IBD directly through perpetuating mucosal inflammation and tissue destruction. Peroxisome proliferator-activated receptor gamma (PPARγ) serves as a crucial regulator of lipid metabolism. In intestinal epithelial cells, PPARγ activation promotes lipocyte differentiation and enhances barrier function by upregulating tight junction proteins [26]. In addition, it exerts potent anti-inflammatory effects by transrepressing NF-κB and AP-1 activity [27,28], thereby suppressing the production of TNF, IL-8, and other inflammatory mediators [29]. Consequently, diminished PPARγ expression or activity observed in IBD patients and experimental colitis models is strongly associated with exacerbated inflammation, impaired healing, and dysbiosis.

It is important to acknowledge that network pharmacology predictions are inherently hypothesis-generating rather than confirmatory, as they rely on existing databases and computational algorithms that cannot replace direct experimental evidence. Similarly, the binding energies obtained from molecular docking only indicate potential compatibility between a compound and its target at a computational level. While they do not reflect true physiological binding affinity or functional engagement in a living system, molecular dynamics simulations are still needed to assess binding stability. Moreover, current computational models are unable to fully capture the dynamic conformational flexibility of proteins or the complex cellular and tissue contexts in which molecular interactions occur. Therefore, future studies must incorporate in vivo validation, for example, using DSS-induced colitis models with assessment of ferroptosis markers, as well as quantitative binding assays such as surface plasmon resonance (SPR) or isothermal titration calorimetry (ITC) to substantiate the predictions.

Emerging evidence has increasingly recognized the gut microbiota as a key regulator of ferroptosis in intestinal inflammatory conditions, and recent studies have begun to elucidate the specific roles of microbiota-derived metabolites in modulating ferroptotic pathways during IBD progression. Zhou et al. comprehensively reviewed the significant roles of gut microbial metabolites—including short-chain fatty acids (SCFAs), tryptophan and its indole derivatives, and bile acids—in ferroptosis regulation during IBD pathogenesis, highlighting that these metabolites influence ferroptosis by modulating the intestinal microenvironment, immune responses, and oxidative stress levels [30]. Gao et al. further systematically examined the interplay between gut microbiota-derived metabolites and ferroptosis, emphasizing the critical role of this interaction in sustaining intestinal homeostasis [31].

Mechanistically, several microbiota-derived metabolites have been identified as direct regulators of ferroptosis in intestinal epithelial cells. Spermidine (SPD), a microbiota-derived polyamine, has been shown to suppress ferroptosis in DSS-induced colitis models by modulating the NCOA4/ferritin axis and stabilizing iron homeostasis, while fecal polyamine metabolites in UC patients were closely correlated with shifts in gut microbial composition [32]. Similarly, the short-chain fatty acid sodium butyrate (NaB), a fermentation product of dietary fiber, alleviated IBD symptoms in mice by inhibiting ferroptosis and modulating the ERK/STAT3 signaling pathway, accompanied by regulation of intestinal flora composition [33]. Notably, a recent study demonstrated that the postbiotic formulation Lacidophilin tablets (LP), derived from Lactobacillus acidophilus, enhanced production of the microbial metabolite 5-hydroxyindoleacetic acid (5-HIAA), which activated the aryl hydrocarbon receptor (AhR) in Lgr5^+^ intestinal stem cells to inhibit ferroptosis and promote epithelial regeneration [34]. Additionally, the dietary flavonoid hispidulin was shown to ameliorate chemically induced colitis by inhibiting epithelial cell ferroptosis via the ACAT2-GPX4 axis while concurrently remodeling the gut microbiota composition [35]. In the context of our study, GAA’s predicted effects on ferroptosis-related pathways may therefore involve, at least in part, interactions with gut microbiota composition and their metabolic outputs.

The findings of this study demonstrate the therapeutic potential of ganoderic acid A (GAA) against IBD. Based on comprehensive KEGG enrichment analyses, the hub genes mentioned above emerged as the most promising candidate targets. This significance stems from their involvement in multiple critical pathways, including lipid metabolism and the mitogen-activated protein kinase (MAPK) signaling cascade. Collectively, these results indicate that GAA may modulate key cellular processes implicated in IBD pathogenesis, encompassing immune modulation, cellular proliferation, and differentiation [36,37]. Molecular docking simulations further provided evidence for several potential molecular targets of GAA relevant to anti-IBD pharmacology. Cytokine/LPS-mediated IBD model in intestinal organoids verified the important role of GAA in IBD alleviation. However, rigorous preclinical evaluation is imperative to establish the safety profile of GAA prior to its consideration for clinical application in IBD.

## 4. Materials and Methods

### 4.1. Data Collection and Processing

Two transcriptomic datasets (GSE38713, GSE126124) were obtained from the GEO database (https://www.ncbi.nlm.nih.gov/geo/, accessed on 18 May 2025). Specifically, the IBD patient-derived colon tissue dataset GSE126124 comprised 21 control samples and 57 colon tissue samples (not whole-blood samples), while GSE38713 had 13 control samples and 15 colon tissue samples from individuals with IBD.

The dataset collection, processing, and normalization were performed using the sva package (Version 3.43.0) in R software (Version 4.5.0). Cross-platform comparability: Both GSE38713 and GSE126124 were generated on the same GPL570 platform (Affymetrix Human Genome U133 Plus 2.0), ensuring identical probe sets and annotation. Therefore, no cross-platform reannotation or transformation was required. Normalization and batch effect correction: Since both datasets shared the same GPL570 platform, we applied ComBat (sva package) to the combined expression matrix, with batch defined as dataset origin (GSE38713 or GSE126124). After batch adjustment, quantile normalization was reapplied across all samples to ensure comparable expression distributions. Assessment of batch correction efficacy: Principal component analysis (PCA) showed that samples clustered primarily by dataset origin before correction but intermingled by disease status afterward, confirming successful removal of technical variation while preserving biological signals.

For identifying the differentially expressed genes (DEGs) between the two groups, the limma package was employed with the screening thresholds set as *p*-values < 0.05 and |logFC| > 0.5. To visualize the expression profiles of DEGs, the pheatmap package and the ggplot2 package were utilized to generate heatmaps and volcano plots, respectively.

### 4.2. Identification of GAA Targets Against IBD

Potential targets of ganoderic acid A (GAA) were retrieved from three pharmacogenomic databases: SwissTargetPrediction (probability threshold > 0.5), ChEMBL (binding affinity IC_50_ < 10 μM), and PharmMapper (fit score > 0.8). Resulting gene lists were harmonized to Homo sapiens gene IDs using DAVID Bioinformatics Resources 6.8. Genes associated with inflammatory bowel disease (IBD) were curated from OMIM and GeneCards (relevance score > 3.0). A total of 458 genes associated with ferroptosis were identified from the ferroptosis database (FerrDb, http://www.zhounan.org/ferrdb/current/ (accessed on 19 May 2025). Intersection analysis of GAA targets, ferroptosis, and IBD-related genes was performed using Venny 2.1 (https://bioinfogp.cnb.csic.es/tools/venny/ (accessed on 19 May 2025) with statistical significance of overlap calculated via Fisher’s exact test (*p* < 0.01).

### 4.3. Weighted Gene Co-Expression Network Analysis (WGCNA)

An input matrix was constructed using all genes from the GSE126124 datasets. WGCNA analysis was performed using the WGCNA package (Version 1.73) in R software. The quality control of data was performed through the goodSamplesGenes function to ensure that there are not too many missing values in the genes, and the variance of gene expression levels is not 0 (or extremely low). Soft thresholding values ranging from 1 to 20 were systematically evaluated to identify the optimal power parameter that approximated a scale-free topology (R^2^ > 0.9). The relationship matrix was transformed into an adjacency matrix, followed by conversion to a topological overlap matrix (TOM) to minimize noise and spurious associations. Average linkage hierarchical clustering of genes based on TOM dissimilarity was performed, with a minimum module size threshold of 50 genes. Modules with high topological overlap (correlation > 0.75) were merged using dynamic tree cutting.

Pearson correlation coefficients were computed between module eigengenes (MEs) and disease status to identify modules most significantly associated with the phenotype. Modules exhibiting the highest positive (ME_Yellow: r = 0.32, *p* < 0.005) and negative (ME_Turquoise: r = −0.33, *p* < 0.004) correlations were designated as core modules. Gene significance (GS) was defined as −log_10_(*p*-value) of the correlation between individual gene expression and disease status, while module membership (MM) was calculated as the correlation between gene expression profiles and the corresponding ME. Genes with high GS (|GS| > 0.15) and MM (|MM| > 0.8) were prioritized as hub candidates (Supplementary Materials S2).

### 4.4. Functional Enrichment Analysis

Functional annotation of candidate genes was performed using the clusterProfiler package (Version 4.8.3) in R software. Concurrently, Kyoto Encyclopedia of Genes and Genomes (KEGG) pathway analysis was employed to map genes to canonical signaling cascades. Both analyses utilized the hypergeometric test with the Benjamini–Hochberg (BH) correction, and terms/pathways with an adjusted *p*-value < 0.05 were considered statistically significant. Visualization of enriched terms was performed using the enrichplot and ggplot2 packages, generating dot plots and bar charts to illustrate hierarchical relationships and gene-pathway associations (Supplementary Materials S2).

### 4.5. Protein–Protein Interaction (PPI) Network Construction

The overlapping gene set was imported into STRING v12.0 with a confidence score threshold of 0.7 (high confidence). The resulting PPI network was exported and analyzed using Cytoscape v3.9.1 to compute node centrality metrics, including degree centrality (DC) and closeness centrality. Core targets were prioritized based on DC values and visualized using the MCODE plugin (score > 5.0) to identify densely connected clusters. Nodes were ranked in descending order of DC and mapped to concentric circles using the Circular Layout algorithm, with node size proportional to DC and edge thickness reflecting interaction confidence.

### 4.6. Gene Set Enrichment Analysis in the IBD Dataset

Gene set enrichment analysis (GSEA) of feature genes from the IBD dataset (GSE126124) was performed using the org.Hs.eg.db annotation package and the clusterProfiler package (Version 4.8.3) in R. The analysis aimed to identify significantly enriched signaling pathways distinguishing IBD from healthy controls, with statistical significance defined as an adjusted *p*-value < 0.05 and a false discovery rate (FDR) < 0.1. To visualize pathway dysregulation, the enrichplot package was employed to generate comprehensive graphics of the most activated and inhibited pathways in IBD (Supplementary Materials S2).

### 4.7. Molecular Docking Assay

The potential binding modes of GAA to its protein targets were investigated using a high-throughput molecular docking approach. Three-dimensional structures of target proteins were retrieved from the Protein Data Bank (PDB) (resolution < 2.5 Å), while the chemical structure of GAA was obtained from PubChem in SDF format. Target proteins were preprocessed using AutoDockTools 1.5.7 to remove water molecules, add polar hydrogens, and compute Gasteiger charges. The ligand was converted to a PDBQT file using Open Babel 3.1.1, with torsional flexibility enabled for all rotatable bonds. The active site of each protein was defined using a grid box centered on the known ligand-binding pocket (grid spacing: 0.375 Å, dimensions: 60 × 60 × 60 points). Molecular docking was performed using AutoDock Vina 1.2.3 with an exhaustiveness parameter of 16. Binding poses were ranked by estimated free energy of binding (ΔGbind) and clustered based on root-mean-square deviation (RMSD < 2.0 Å). Docking results were visualized using PyMOL 2.3.

### 4.8. Culture of Intestinal Organoids and Cell Viability Assay

The kit for inducing differentiation of intestinal organoids (hiPSCs) using pluripotent stem cells was purchased from Huante Biotechnology Co., Ltd. (Hangzhou, China). HiPSCs were seeded at a density of 1 × 10^5^ cells per well in a 6-well plate coated with Matrigel for revival culture. 3 mL of mTeSR Plus culture medium containing 10 μmol/L Y-27632 was added to each well, and the medium was changed daily until the fusion degree reached 80–90%. The original culture medium was discarded, and 1 mL of DPBS was added at 37 °C to each well to wash the cells. Digestion was performed with ReLeSR cell digestion solution for 1 min, followed by incubation in a 5% CO_2_ incubator at 37 °C for 5–6 min. After that, 1 mL of culture medium was added, and subculture was performed at a ratio of 1:20. The growth of organoids was observed until the formation of complex budding structures on day 4.

Characterization of intestinal organoids:

Differentiation efficiency and organoid maturation were assessed by morphological observation and marker expression analysis. Following the manufacturer’s protocol, hiPSCs were sequentially directed toward definitive endoderm, mid/hindgut endoderm, and intestinal epithelium using the commercial differentiation kit (Huante Biotechnology Co., Ltd., Hangzhou, China). This stepwise approach recapitulates critical developmental stages of human intestinal morphogenesis. Organoid growth was monitored daily under an inverted microscope. On days 10–14 post-seeding, the formation of complex budding structures with crypt-like domains was observed, indicative of successful intestinal organoid development. To confirm intestinal epithelial identity, immunofluorescence staining was performed on frozen sections of d14 organoids. Organoids were fixed with 4% paraformaldehyde, permeabilized, and blocked with 5% BSA. Primary antibodies against the intestinal epithelial markers Villin, E-cadherin, and the stem cell marker LGR5 were applied, followed by appropriate Alexa Fluor-conjugated secondary antibodies. Only organoid preparations exhibiting robust crypt budding and positive expression of the above markers were used for subsequent experiments to ensure model reproducibility, following established quality assessment guidelines.

Cell viability was assessed using the MTT assay, as previously described with modifications. Briefly, cells were seeded in 96-well culture plates at a density of 5 × 10^3^ cells per well in 100 µL of complete growth medium and allowed to adhere overnight under standard culture conditions (37 °C, 5% CO_2_). After treatment, 10 µL of MTT reagent (5 mg/mL in phosphate-buffered saline (PBS)) was added to each well, and the plates were incubated for an additional 4 h at 37 °C. The optical density (OD) of the resulting solution was measured at a wavelength of 570 nm, with a reference wavelength of 630 nm to correct for non-specific absorption.

### 4.9. Construction of the IBD Model in Intestinal Organoids and GAA Treatment

To induce inflammation in organoids to simulate inflammatory bowel disease, the following drugs were continuously applied to organoids cultured for 7 days: 100 ng/mL LPS, 10 ng/mL IL-1β, and 10 ng/mL TNF-α (TNF-α group) [38]. The complete medium was replaced every 48 h. The organoids in the model group exhibited obvious shrinkage and darkening morphology, which were considered IBD models at day 7. The control group was treated with PBS, while the GAA group was co-treated with 60 µM GAA for 24 h after IBD modeling [22].

### 4.10. Western Blot Analysis

Western blot analysis was performed according to a previous study. Primary antibodies: glyceraldehyde-3-phosphate dehydrogenase (GAPDH), tumor necrosis factor α (TNF-α), phospho-mitogen-activated protein kinase 14 (p-MAPK14), and peroxisome proliferators-activated receptor γ (PPARγ) were purchased from Abcam (Waltham, MA, USA). HRP-conjugated anti-rabbit IgG was purchased from Abclone (Boston, MA, USA).

### 4.11. EdU Staining Assay

Cell proliferation was assessed using a commercial EdU assay kit (EdU Alexa Fluor™ 594 Imaging Kit, Thermo Fisher Scientific, Waltham, MA, USA) according to the manufacturer’s instructions, with minor modifications. Briefly, cells in the logarithmic growth phase were seeded into confocal dishes at an appropriate density and allowed to adhere overnight. After the respective treatments, the culture medium was replaced with fresh medium containing 10 µM EdU reagent, and cells were incubated for 2 h at 37 °C in a 5% CO_2_ incubator to allow for the incorporation of EdU into newly synthesized DNA.

Following incubation, the cells were washed twice with PBS and fixed with 4% paraformaldehyde for 15 min at room temperature. After fixation, the cells were permeabilized with 0.5% Triton X-100 for 20 min at RT and washed again twice with PBS. The reaction mixture containing Alexa Fluor™ 594 azide, reaction buffer, and additive was added to each sample and incubated for 30 min at RT and protected from light. Images were captured using a laser scanning confocal microscope under consistent exposure settings for all groups within the same experiment. The percentage of EdU-positive cells (proliferation rate) was quantified by counting the number of red fluorescent (EdU-positive) nuclei using ImageJ, version 1.54. The proportion of EdU-positive cells was determined by dividing the number of EdU+ nuclei by the total number of DAPI+ nuclei.

### 4.12. Statistical Analysis

All statistical analyses were performed using SPSS version 22.0 (SPSS Inc., Chicago, IL, USA), and values are expressed as the mean ± SEM, unless otherwise specified. The significance of the differences between all groups was analyzed by one-way ANOVA or *t*-test. *p*-value < 0.05 was considered significant, and *p*-value < 0.01 was considered very significant.

## 5. Conclusions

Based on integrated network pharmacology, molecular docking analysis, and intestinal organoid validation, GAA may exert multi-target effects on immune homeostasis and lipid metabolism-related pathways in IBD. Our computational analysis also identified a potential intersection with ferroptosis-associated gene sets, but this finding is preliminary and lacks experimental verification. GAA demonstrates high-affinity binding to core targets, suppressing inflammation and epithelial barrier disruption. Crucially, experimental evaluation using a cytokine/LPS-induced IBD model in intestinal organoids demonstrated the pivotal role of GAA in alleviating disease pathology. These findings position GAA as a promising functional food-derived therapeutic candidate for IBD, warranting further preclinical validation of its translational potential.

## Figures and Tables

**Table 1:** Identification of overlapping genes identified as potential targets of GAA in the context of IBD.

Genes
PPARγ, TNF, CASP3, mTOR, EGFR, PPARα, SRC, MAPK14, PIK3CA, ADIPOQ, AR, NOS2, G6PD, ALOX5

**Table 2:** The 54 WGCNA-derived genes.

Down-Regulated Genes (Turquoise Module)
MAPK14, IER5, RAB3GAP2, KDM2A, TAS2R31, MED13L, COIL, BIRC6, TNF, NBAS, RALGAPA2, FAM172A, TRRAP, WRN, NFIB
Up-regulated genes (Yellow module)
PIN1P1, MEF2D, SEC24C, NDUFB8, NDUFS8, PIK3CA, BAD, APAF1, BCL7A, PPARγ, MIR134, ATG2B, BRF1, NUDT16L1, HSF4, PYCARD, ANAPC11, OXLD1, PQLC1, RPL36, CASP3, DHDH, NDUFA7, KLF1, TRAPPC6A, HMGN2P20, OR7E31P, DBNDD2, RN7SKP74, TMEM50B, DDTL, ATP11B, TUSC2, HIGD2A, ARF5, MIR182, MRPL41, ERP44, NGFRAP1

## Data Availability

The data that support the findings of this study are available from the corresponding author upon reasonable request.

## Supplementary Materials

The following supporting information can be downloaded at: https://www.mdpi.com/article/10.3390/ijms27135698/s1.

## Abbreviations

The following abbreviations are used in this manuscript:

AE	Aqueous extract
GAPDH	Glyceraldehyde-3-phosphate dehydrogenase
TNF	Tumor necrosis factor
KEGG	Kyoto Encyclopedia of Genes and Genomes
