# Supplementary material for: Exploring the Therapeutic Potential of Ganoderic Acid A Against Inflammatory Bowel Disease Based on Network Pharmacology, Molecular Docking, and Intestinal Organoid Validation
Source: Int J Mol Sci. 2026 Jun 24;27(13):5698. doi: 10.3390/ijms27135698 (PMC13361695; doi:10.3390/ijms27135698)
Supplement: Supplementary file 1 [file ijms-27-05698-s001.zip › S2_R scripts.pdf]

```
library(AnnoProbe)
library(tidyverse)
library(tinyarray)
library(sva)
library(FactoMineR)
library(factoextra)
library(limma)
library(dplyr)

file_list <- c("DATA1.txt", "DATA2.txt", "DATA3.txt","DATA4.txt")
sample_prefixes <- c("CGGA", "GSM", "AK","BAK")
batch_labels <- c("GSE126123", "GSE38713")
pca_colors <- c("#00AFBB", "#E7B800", "#FC4E07", "#2E9FDF", "#E74C3C")
output_combined_file <- "mergefile.txt"

data_list <- list()
gene_cols <- list()

for (i in 1:length(file_list)) {
  data <- read.table(file_list[i], header=TRUE, sep="\t", check.names=FALSE,
row.names=1)
      data$gene <- rownames(data)
  data_list[[i]] <- data
  gene_cols[[i]] <- data$gene
}
combined_data <- data_list[[1]]

for (i in 2:length(data_list)) {
  combined_data <- inner_join(combined_data, data_list[[i]], by = "gene")
}
combine_data <- combined_data %>% select(-gene) %>% as.data.frame()
rownames(combine_data) <- combined_data$gene

batch <- c()
for (i in 1:length(data_list)) {
      sample_names <- colnames(data_list[[i]])[colnames(data_list[[i]]) != "gene"]
  batch <- c(batch, rep(batch_labels[i], length(sample_names)))
}
pdf(file = "merge.pdf")
boxplot(combine_data, outline=FALSE, notch=FALSE, las=2, xaxt="n")
dev.off()
combine_after_data <- ComBat(dat=as.matrix(combine_data), batch=batch,
mod=NULL, par.prior=TRUE)
```

```
combine_after_data <- normalizeBetweenArrays(combine_after_data)

write.table(combine_after_data, output_combined_file, sep="\t", quote=FALSE, row.names = TRUE)

library(limma)
library(pheatmap)
library(ggplot2)
library(EnhancedVolcano)

FPKM <- read.table("260401log_tpms.txt", header=TRUE, sep="\t", check.names=FALSE)

geneNames <- FPKM[,1]
expMatrix <- FPKM[,-1]

expMatrix <- as.data.frame(lapply(expMatrix, function(x) as.numeric(as.character(x))))
expMatrix[is.na(expMatrix)] <- 1e-6

normalizeBetweenArrays
rownames(expMatrix) <- geneNames

sample_info <- read.table("design4.txt", header=TRUE, sep="\t", row.names=1, stringsAsFactors=FALSE)

if(is.vector(sample_info)) {
  sample_info <- as.data.frame(sample_info)
  colnames(sample_info) <- "group"
}

group_list <- factor(sample_info$group, levels=c("Control", "F2"))

log_TPM <- log2(expMatrix + 1)

pdf("Boxplot_log_TPM.pdf")
boxplot(log_TPM, outline=FALSE, col=group_list, las=2, main="Log2(TPM+1) Distribution")
dev.off()

design <- model.matrix(~0 + group_list)
colnames(design) <- c("Control", "F2")

fit <- lmFit(log_TPM, design)
```

```
contrast.matrix <- makeContrasts(F2 - Control, levels=design)
fit2 <- contrasts.fit(fit, contrast.matrix)
fit2 <- eBayes(fit2, trend=TRUE)

deg <- topTable(fit2, adjust='BH', number=Inf)

hist(deg$logFC, main="Distribution of logFC", xlab="logFC")

Deg_gene <- subset(deg, adj.P.Val < 0.05 & abs(logFC) >= 0.25)

write.csv(deg, file="All_DEGs_results.csv")
write.table(Deg_gene, file="Significant_DEGs.csv", sep=",", col.names=NA,
quote=FALSE)

library(DOSE)
library(clusterProfiler)
library(org.Hs.eg.db)
library(dplyr)
library(GOplot)
library(tidyverse)
library(viridis)
library(DOSE)
library(ggplot2)
library(tidyr)

file_path <- "All_DEGs_results.txt"

table_data <- read.table(file_path, sep = "\t", header = TRUE, check.names = FALSE)

gene_column <- table_data[[1]]
entrez_ids <- c()

for (gene in gene_column) {
    id <- tryCatch({
        mget(gene, org.Hs.egSYMBOL2EG)
  }, error = function(e) {
        NA
  })

    entrez_ids <- c(entrez_ids, id)
}

entrez_ids <- as.character(entrez_ids)
```

```
table_data <- cbind(table_data, entrezID = entrez_ids)
table_data <- table_data[!is.na(table_data[, "entrezID"]), ]

gene <- table_data$entrezID
KEGG <- enrichKEGG(gene = gene,
                   organism = "hsa",
                   pvalueCutoff = 1,
                   qvalueCutoff = 1)

KEGGG <- as.data.frame(KEGG)
KEGGG <- KEGGG[, !names(KEGGG) %in% "category"]

names(KEGGG)[names(KEGGG) == "subcategory"] <- "ONTOLOGY"
KEGGG$ONTOLOGY <- "KEGG"
KEGG2 <- KEGG
KEGG2@result <- KEGGG
write.table(KEGG, file = "KEGG.txt", sep = "\t", quote = FALSE, row.names = FALSE)
interest_terms2 <- c("hsa05323", "hsa04514", "hsa00100",
                     "hsa04512", "hsa04640" )

selected_GO2 <- subset(KEGG2, KEGG2$ID %in% interest_terms2)
KEGG2@result <- selected_GO2
pdf("KEGG-bubble.pdf", width = 13, height = 10)


library(clusterProfiler)

library(enrichplot)

library(ggplot2)

need_DEG <- read.csv("log2FC.csv",row.names = 1)

colnames(need_DEG) <- c('log2FoldChange')

need_DEG$SYMBOL <- rownames(need_DEG)



geneList <- need_DEG$log2FoldChange

names(geneList) <- need_DEG$SYMBOL

geneList <- sort(geneList, decreasing = T)
```

```
geneset <- read.gmt("c2.cp.kegg.v2023.1.Hs.symbols.gmt")

KEGG_GSEA <- GSEA(

  geneList = geneList,

  TERM2GENE = geneset,

  #verbose = F,

  minGSSize = 1,

  maxGSSize = 100000,

  eps=1e-10,

  pvalueCutoff = 1,

  pAdjustMethod = "BH"#“holm”, “hochberg”, “hommel”, “bonferroni”, “BH”,
“BY”, “fdr”, “none”

)


kegg_gsea <- data.frame(KEGG_GSEA@result)

write.csv(kegg_gsea,"KEGG_GSEA_result.csv")


i = 6

p <- gseaplot2(KEGG_GSEA, geneSetID =i,

                    title = KEGG_GSEA$Description[i])

p

ggsave("kegg_gsea.pdf",p,width = 6,height = 4.5)
```

```
library(WGCNA)

library(flashClust)

library(iterators)

library(dplyr)

library(ff)


datExpr0 <- read.csv("exprSet.csv",row.names = 1)

datExpr0 <- data.frame(t(datExpr0 ))



gsg <- goodSamplesGenes(datExpr0, verbose = 3)

gsg$allOK

if(!gsg$allOK){

 # if(sum(!gsg$goodGenes)>0)

   #    printFlush(paste("Removinggenes:",paste(names(datExpr0)[!gsg$goodGenes],
collapse =",")));

 # if(sum(!gsg$goodSamples)>0)

   #    printFlush(paste("Removingsamples:",paste(rownames(datExpr0)[!gsg$goodSa
mples], collapse =",")));

   datExpr0 = datExpr0[gsg$goodSamples, gsg$goodGenes]

}

group <- read.csv('group.csv', row.names = 1, check.names = F)

group$disease <- ifelse(group$group == 'disease', 1, 0)

group$control <- ifelse(group$group == 'control', 1, 0)

group <- dplyr::select(group, -group)

group <- plyr::rename(group,
```

```
                              c(sample = 'id'))
datTraits <- data.frame(row.names = group$id, group = group[, 2:3])


sampleTree = hclust(dist(datExpr0), method ="average");
par(cex = 0.6)
par(mar =c(0,4,2,0))
plot(sampleTree, main ="Sample clustering to detectoutliers",sub="", xlab="", cex.lab
= 1.5,
     cex.axis= 1.5, cex.main = 2)
clust = cutreeStatic(sampleTree, cutHeight = 80, minSize = 10)
table(clust)
keepSamples <- (clust == 1)
datExpr <- datExpr0[keepSamples, ]
nGenes <- ncol(datExpr)
nSamples <- nrow(datExpr)


load("GSE64634_eSet.Rdata")
exp <- exprs(GEO_file[[1]])
plate <- fData(GEO_file[[1]])
clinical <- pData(GEO_file[[1]])
write.csv(clinical,"ClinicalTraits.csv")
traitData <- read.csv("ClinicalTraits.csv")
allTraits <- traitData[, -c(31, 16)]
allTraits <- traitData[,c(2, 11:36) ]
```

```
datTraits <- allTraits

rownames(traitData) <- traitData$sample

datTraits <- datTraits[rownames(datExpr),]

datTraits <- traitData[-1]

#collectGarbage()

sampleTree2 = hclust(dist(datExpr), method ="average")

traitColors = numbers2colors(datTraits, signed = FALSE);

plotDendroAndColors(sampleTree2,

                    traitColors,

                    groupLabels = names(datTraits),

                    main ="Sample dendrogram and trait heatmap")


powers <- c(c(1:10), seq(from = 12, to=20, by=2))
sft <- pickSoftThreshold(datExpr, powerVector = powers, verbose = 5)
sizeGrWindow(9, 5)
par(mfrow = c(1, 2));
cex1 = 0.9;
plot(sft$fitIndices[, 1],
     -sign(sft$fitIndices[, 3])*sft$fitIndices[, 2],
     xlab = "Soft Threshold (power)",
     ylab = "Scale Free Topology Model Fit,signed R^2",
     type = "n",
     main = paste("Scale independence"));
text(sft$fitIndices[, 1],
     -sign(sft$fitIndices[, 3])*sft$fitIndices[, 2],
     labels = powers,
     cex = cex1,
     col = "red");
abline(h=0.85,col="red")
plot(sft$fitIndices[, 1],
     sft$fitIndices[, 5],
```

```
        xlab = "Soft Threshold (power)",
        ylab = "Mean Connectivity",
        type = "n",
        main = paste("Mean connectivity"))
text(sft$fitIndices[, 1],
        sft$fitIndices[, 5],
        labels = powers,
        cex = cex1,
        col = "red")



datExpr <- read.csv.ffdf(file = "datExpr.csv")


nGenes = ncol(datExpr)
nSamples = nrow(datExpr)

net <- blockwiseModules(datExpr,
                        power = 6,
                        maxBlockSize = nGenes,
                        TOMType = "unsigned",
                        minModuleSize = 50,
                        reassignThreshold = 0,
                        # deepSplit = 2,
                        mergeCutHeight = 0.3,
                        numericLabels = TRUE,
                        pamRespectsDendro = FALSE,
                        saveTOMs = TRUE,
                        saveTOMFileBase = "gene_TOM",
                        corType = "pearson",
                        loadTOMs = TRUE,
                        verbose = 3)

table(net$colors)
mergedColors <- labels2colors(net$colors)

sizeGrWindow(12, 9)
plotDendroAndColors(net$dendrograms[[1]],
                    mergedColors[net$blockGenes[[1]]],
                    "Module colors",
                    dendroLabels = FALSE,
                    hang = 0.03,
                    addGuide = TRUE,
                    guideHang = 0.05)
```

```
moduleLabels <- net$colors
moduleColors <- labels2colors(net$colors)
table(moduleLabels)
table(moduleColors)

## Recalculate MEs with color labels
MEs0 <- moduleEigengenes(datExpr, moduleColors)$eigengenes
MEs <- orderMEs(MEs0)

datTraits <- plyr::rename(datTraits,
                          c(group.disease = 'disease',
                            group.control = 'control'))
MEs <- dplyr::select(MEs, -MEgrey)
moduleTraitCor = cor(MEs, datTraits, use = "p");
moduleTraitPvalue <- corPvalueStudent(moduleTraitCor, nSamples)

textMatrix <- paste(signif(moduleTraitCor, 2), "\n(",
                    signif(moduleTraitPvalue, 1), ")", sep = "");
dim(textMatrix) <- dim(moduleTraitCor)

par(mar = c(4, 9, 2, 2))
labeledHeatmap(Matrix = moduleTraitCor,
               xLabels = names(datTraits),
               yLabels = names(MEs),
               ySymbols = names(MEs),
               colorLabels = FALSE,
               colors = blueWhiteRed(50),
               textMatrix = textMatrix,
               setStdMargins = FALSE,
               cex.text = 0.7,
               zlim = c(-1,1),
               main = paste("Module-trait relationships"))


module <- c('yellow')
probes <- colnames(datExpr)
inModule <- (moduleColors == module)
modProbes <- probes[inModule]
modGenes <- as.data.frame(modProbes)
colnames(modGenes) <- 'modgene'

geneModuleMembership <- as.data.frame(cor(datExpr, MEs, use = "p"))
```

```
MMPvalue <- as.data.frame(corPvalueStudent(as.matrix(geneModuleMembership),
nSamples))

geneTraitCor = as.data.frame(cor(datExpr, datTraits, use = "p"))
geneTraitP = as.data.frame(corPvalueStudent(as.matrix(geneTraitCor), nSamples))

modNames = substring(colnames(MEs), 3)

pheno = "disease"
module_column = match(module, modNames)
pheno_column = match(pheno, colnames(datTraits))

moduleGenes <- (moduleColors == module)

MM <- abs(geneModuleMembership[moduleGenes, module_column])
GS <- abs(geneTraitCor[moduleGenes, 1])

dev.new(width=10, height=7)
par(mar = c(4, 4, 3, 2))
verboseScatterplot(x = MM,
                   y = GS,
                   xlab = paste("Module Membership in", module, "module"),
                   ylab = paste("Gene significance for", pheno),
                   main = paste("Module membership vs. gene significance\n"),
                   cex.main = 1.2, cex.lab = 1.2, cex.axis = 1.2, col = module)
abline(h=0.6,v=0.8,col="blue",lwd=1.5)

c <- as.data.frame(cbind(MM, GS))
rownames(c) <- modGenes$modgene

hub_gene <- subset(c, c$MM > 0.8 & c$GS > 0.6)
write.csv(hub_gene, file = paste0(module, '_screen_gene_GS_MM.csv'))
```
